# Adenosine A_2A_ Receptor Occupancy by Caffeine After Coffee Intake in Parkinson's Disease

**DOI:** 10.1002/mds.28897

**Published:** 2022-01-09

**Authors:** Kenji Ishibashi, Yoshiharu Miura, Kei Wagatsuma, Jun Toyohara, Kiichi Ishiwata, Kenji Ishii

**Affiliations:** ^1^ Research Team for Neuroimaging Tokyo Metropolitan Institute of Gerontology Tokyo Japan; ^2^ Department of Neurology Tokyo Metropolitan Cancer and Infectious Diseases Center Komagome Hospital Tokyo Japan; ^3^ School of Allied Health Science Kitasato University Sagamihara Japan; ^4^ Institute of Cyclotron and Drug Discovery Research Southern Tohoku Research Institute for Neuroscience Koriyama Japan; ^5^ Department of Biofunctional Imaging Fukushima Medical University Fukushima Japan

**Keywords:** adenosine A_2A_ receptor, coffee, caffeine, Parkinson's disease, ^11^C‐preladenant PET

## Abstract

**Background:**

Coffee intake can decrease the risk for Parkinson's disease (PD). Its beneficial effects are allegedly mediated by caffeine through adenosine A_2A_ receptor (A_2A_R) antagonist action.

**Objective:**

We aimed to calculate occupancy rates of striatal A_2A_Rs by caffeine after coffee intake in PD.

**Methods:**

Five patients with PD underwent ^11^C‐preladenant positron emission tomography scanning at baseline and after intake of coffee containing 129.5 mg (n = 3) or 259 mg (n = 2) of caffeine. Concurrently, serum caffeine levels were measured.

**Results:**

The mean serum caffeine level (μg/mL) was 0.374 at baseline and increased to 4.48 and 8.92 by 129.5 and 259 mg of caffeine, respectively. The mean occupancy rates of striatal A_2A_Rs by 129.5 and 259 mg of caffeine were 54.2% and 65.1%, respectively.

**Conclusions:**

A sufficient A_2A_R occupancy can be obtained by drinking a cup of coffee, which is equivalent to approximately 100 mg of caffeine. © 2022 The Authors. *Movement Disorders* published by Wiley Periodicals LLC on behalf of International Parkinson and Movement Disorder Society.

Epidemiological studies have consistently demonstrated that coffee intake can decrease the risk for development of Parkinson's disease (PD).^1^, ^2^ Because decaffeinated coffee is not protective against PD,^1^ caffeine in coffee is believed to be the essential pharmacological factor contributing to its beneficial effects in PD. Caffeine mainly works as a nonselective blocker of all four adenosine receptor subtypes: A_1_ (K_D_ = 12 μM), A_2A_ (K_D_ = 2.4 μM), A_2B_ (K_D_ = 13 μM), and A_3_ (K_D_ = 80 μM).^3^ Of these subtypes, adenosine A_2A_ receptors (A_2A_Rs) are believed to underlie most of the beneficial effects of caffeine in PD,^4^ although its mechanism is unclear.

A_2A_Rs are predominantly distributed in the putamen, caudate, nucleus accumbens, and external globus pallidus, and they interact with dopamine D_2_ receptors in the indirect basal ganglia pathway.^5^, ^6^, ^7^ Because of these features, A_2A_Rs have been recognized as a therapeutic target to modulate motor symptoms in PD. Istradefylline, a selective A_2A_R antagonist (K_i_ = 12.4 nM),^8^ was then launched in Japan in 2013 as an adjunct to levodopa to alleviate *off* episodes in PD and was subsequently approved by the US Food and Drug Administration in 2019.^9^, ^10^ Currently, once‐daily oral administration of istradefylline 20 or 40 mg is recommended. We found that occupancy rates of striatal A_2A_Rs after single administration of istradefylline 20 and 40 mg were 39.5% and 52.1%, respectively,^11^ and that the corresponding rates after long‐term administration of istradefylline 20 and 40 mg increased to 72.1% and 86.5%, respectively.^12^

We hypothesized that if the beneficial effects of coffee in PD are mediated by caffeine through A_2A_R antagonist action, a substantial amount of A_2A_R should be occupied by caffeine after coffee intake, similar to that observed after administration of istradefylline.^11^, ^12^ This study aimed to test the hypothesis by calculating occupancy rates of striatal A_2A_Rs after coffee intake in patients with PD using ^11^C‐preladenant positron emission tomography (PET) for measurement of A_2A_R availability. Concurrently, the amount of caffeine in coffee was verified, and the serum caffeine levels were measured.

## Materials and Methods

### Research Participants

This study was conducted in accordance with the Declaration of Helsinki and approved by the Ethics Committee of the Tokyo Metropolitan Institute of Gerontology (R19‐19). Written informed consent was obtained from all five patients with PD (three men and two women) aged 61 to 76 years (Table 1). All patients were taking at least one antiparkinsonian drug other than istradefylline. Two patients regularly consumed at least one cup of coffee per day. Two other patients occasionally drank coffee. The remaining one patient rarely drank coffee. None of the patients had a history of smoking after middle age.

**TABLE 1:** Characteristics of the patients with Parkinson's disease

	Patient No.				
Characteristics	1	2	3	4	5
Dose of caffeine, mg	259	259	129.5	129.5	129.5
Age, y	74	61	71	69	61
Sex	Male	Male	Male	Female	Female
Weight, kg	60.1	47.5	61.5	52.3	48.2
Coffee consumption	1 cup/day	1 cup/day	Occasionally	Occasionally	Rarely
Duration, y	6	13	3	5	3
Hoehn & Yahr stage	3	3	3	3	2
Medication	l‐Dopa pramipexole	l‐Dopa pramipexole	l‐Dopa	l‐Dopa pramipexole	Ropinirole
Serum caffeine level, μg/mL					
Baseline	0.56	0.29	0.06	0.96	0.00
Caffeine loading	8.94	8.90	4.51	4.58	4.36
BP_ND_ in the striatum^a^					
Baseline	4.34	3.89	3.16	3.06	4.16
Caffeine loading	1.68	1.21	1.50	1.42	1.79
Occupancy, %	61.3	68.9	52.4	53.4	57.0

**FIG 1:**
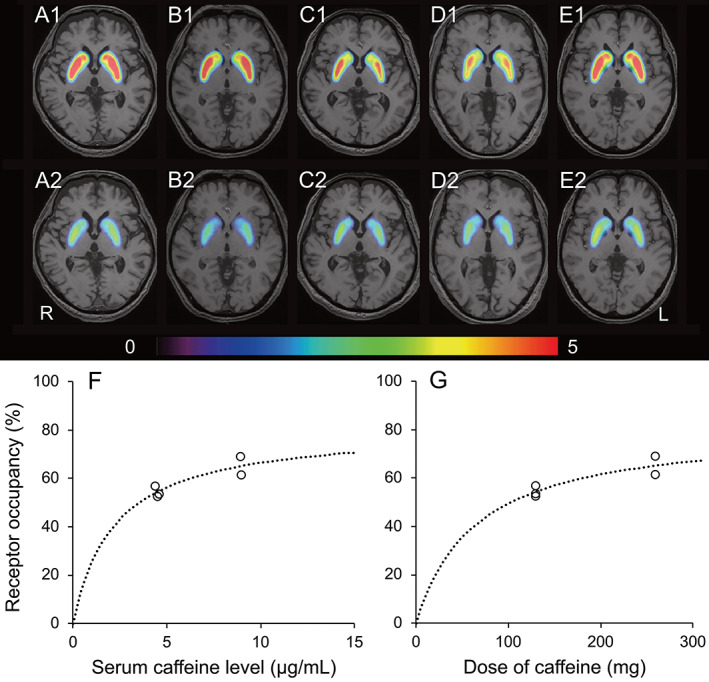
Changes in binding potential (BP_ND_) maps after caffeine intake in five patients with Parkinson's disease (**A**–**E**) and the relationships between adenosine A_2A_ receptor occupancy and serum caffeine levels (**F**) or caffeine dose (**G**). BP_ND_ maps of adenosine A_2A_ availability in patients 1 (A), 2 (B), 3 (C), 4 (D), and 5 (E) are displayed on structural magnetic resonance imaging as follows: at baseline (A1, B1, C1, D1, and E1) and after intake of coffee containing 259 mg (A2 and B2) or 129.5 mg (C2, D2, and E1) of caffeine. The rainbow‐colored scale represents the magnitude of BP_ND_ values. Patients 1–5 correspond to the numbers of the five patients in Table 1. The dashed curve was modeled using the following equation: occupancy (%) = α × [D/(D + ED_50_)], where α refers to the maximal receptor occupancy, D refers to serum caffeine levels (**F**) or caffeine dose (**G**), and ED_50_ refers to the level resulting in 50% of maximal receptor occupancy. L, left; R, right. [Color figure can be viewed at wileyonlinelibrary.com]

Patients 1–5 correspond to Fig. 1A–E, respectively.

l‐Dopa, levodopa.

^a^ Binding potential (BP_ND_) was calculated using the volume‐of‐interest‐based method.^11^

### Study Protocol, Coffee Intake, and Serum Caffeine Level

Commercially available “canned coffee” (Suntory Premium Boss; Suntory Holdings Limited, Osaka, Japan) was used for caffeine loading by drinking coffee. One can of coffee (185 g) contained 129.5 mg of caffeine (70 mg/100 g). The five patients with PD were classified into either the low‐caffeine‐loading group or the high‐caffeine‐loading group according to their requests. The two patients in the high‐caffeine‐loading group consumed two cans of coffee containing 259 mg of caffeine. The other three patients in the low‐caffeine‐loading group consumed one can of coffee containing 129.5 mg of caffeine. To calculate A_2A_R occupancy rates by caffeine after coffee intake, each patient underwent a total of two ^11^C‐preladenant PET scans in two conditions: caffeine restricted (ie, baseline) and caffeine loading. The interval between the two PET scans was less than 3 months.

In both caffeine‐restricted and caffeine‐loading conditions, all patients were instructed to avoid consuming caffeine‐containing products such as coffee, tea, energy drinks, or chocolate from the evening before undergoing PET. We also instructed them not to withhold any antiparkinsonian drugs before the PET scan. On the day of the examination, all patients visited the PET center after taking breakfast and their antiparkinsonian drugs. In the caffeine‐restricted condition, the PET scan began at about 13:00 after blood samples were collected immediately before the injection of ^11^C‐preladenant. In the caffeine‐loading condition, each patient drank one can or two cans of coffee at about 12:15. The PET scan was then started at about 13:00 immediately after the collection of blood samples.

The collected blood samples were transferred to the Tsukuba Research Institute (BoZo Research Center, Tokyo, Japan), and serum caffeine levels were measured using liquid chromatography mass spectrometry. The pharmacokinetics of caffeine have been established.^13^ After oral administration of a single 250‐mg dose of caffeine, the peak plasma caffeine level reaches approximately 10 μg/mL in an hour, and the plasma elimination half‐lives (t_1/2_) range from 3 to 7 hours. Therefore, the ^11^C‐preladenant PET scan in the caffeine‐loading condition was performed approximately 45 minutes after coffee intake; as such, serum caffeine level reached a peak during PET scanning.

### PET and Data Analysis

^11^C‐preladenant PET scanning and image processing were conducted basically as described previously.^11^, ^12^ In brief, after a bolus injection of approximately 500 MBq of ^11^C‐preladenant, emission data were acquired for 60 minutes. Binding potential (BP_ND_) in the whole striatum was calculated to measure A_2A_R availability using the Simplified Reference Tissue Model,^14^ after the cerebellum was set as a reference region. In addition, BP_ND_ maps were generated using the Simplified Reference Tissue Model 2.^15^

The A_2A_R occupancy was calculated using the following equation: Occupancy (%) = 100 × [(BP_ND_ in caffeine restricted) − (BP_ND_ in caffeine loading)]/(BP_ND_ in caffeine restricted). The relationships between A_2A_R occupancy and serum caffeine levels (μg/mL) or dose of caffeine (mg) were modeled using the following equation: occupancy (%) = α × [D/(D + ED_50_)], where α refers to the maximal receptor occupancy, D refers to serum caffeine levels or dose of caffeine, and ED_50_ refers to the level resulting in 50% of maximal receptor occupancy.^16^, ^17^, ^18^ The two parameters, α and ED_50_, were estimated with a nonlinear regression analysis, using SPSS Statistics version 25 (IBM Corporation, Armonk, NY, USA).

## Results

The serum caffeine levels and striatal BP_ND_ values are shown in Table 1. The mean serum caffeine level (μg/mL) was 0.374 in the caffeine‐restricted condition (n = 5) and increased to 4.48 and 8.92 in the low‐caffeine‐loading (129.5 mg: n = 3) and high‐caffeine‐loading (259 mg: n = 2) conditions, respectively. The mean A_2A_R occupancy rates in the low‐caffeine and high‐caffeine groups were 54.2% and 65.1%, respectively. The changes in the striatal BP_ND_ values after coffee intake are displayed in BP_ND_ maps (Fig. 1A–E).

The relationships between A_2A_R occupancy and serum caffeine levels or doses of caffeine are depicted (Fig. 1F,G). The two estimated parameters, maximal receptor occupancy and ED_50_, in the relationship between A_2A_R occupancy and serum caffeine levels were 81.1% (standard error [SE], 10.0%) and 2.2 μg/mL (SE, 1.0 μg/mL), respectively. The corresponding values in the relationship between A_2A_R occupancy and dose of caffeine were 81.3% (SE, 9.4%) and 64.7 mg (SE, 27.2 mg), respectively.

## Discussion

This study found that the mean occupancy rates of striatal A_2A_Rs by 129.5 and 259 mg of caffeine were 54.2% and 65.1%, respectively. Meanwhile, the corresponding rates after single administration of istradefylline 20 and 40 mg are 39.5% and 52.1%, respectively,^11^ and those after long‐term administration of istradefylline 20 and 40 mg are 72.1% and 86.5%, respectively.^12^ These findings suggest that striatal A_2A_R occupancy by caffeine after coffee intake is comparable with that by the administration of the approved dose of istradefylline (20–40 mg) and strongly support the hypothesis that a substantial amount of A_2A_R is occupied by caffeine after coffee intake.

Zhou et al.^19^ assessed the suitability of ^11^C‐preladenant PET for the quantification of striatal A_2A_Rs and calculated occupancy rates of striatal A_2A_R by caffeine in conscious monkeys. They demonstrated that occupancy rates after intravenous injections of caffeine at doses of 2.5, 5.0, and 10.0 mg/kg were 64%, 74%, and 81%, respectively. Meanwhile, the average body weight of five patients in our study was 54 kg. Applying this mean value to 129.5 and 259 mg of caffeine, our results restated that occupancy rates of striatal A_2A_Rs after administration of 2.4 and 4.8 mg/kg caffeine were 54.2% and 65.1%, respectively. Given the methodological differences, our results from human patients seem to agree well with those carried out by Zhou et al.^19^ involving animals.

The ED_50_ values were estimated to be 2.2 μg/mL for serum caffeine levels and 64.7 mg for doses of caffeine. A cup of coffee generally contains approximately 100 mg of caffeine. Therefore, after drinking a cup of coffee (ie, intake of 100 mg of caffeine), serum caffeine level can exceed its ED_50_ value (ie, 2.2 μg/mL). According to the National Coffee Association, USA, the average American coffee drinker drinks about three cups per day, which is equivalent to approximately 300 mg of caffeine per day. Considering the ED_50_ (64.7 mg) and t_1/2_ (3–7 hours) of caffeine, it is quite possible that at least approximately 50% of striatal A_2A_Rs is constantly blocked by caffeine in coffee drinkers. This estimated occupancy rate is novel and is an important finding to help understand the impact of caffeine on health and disease in coffee drinkers.^4^, ^20^

Recently, a randomized controlled trial reported that compared with administration of placebo, consumption of caffeine‐containing capsules 200 mg twice daily did not improve motor manifestations in PD.^21^ This result appears to conflict with epidemiological links between coffee intake and lower PD risk.^1^, ^2^ One possible reason for this contradiction is that in the placebo group, daily intake of 92.17 ± 50.30 mg of caffeine was allowed during the trial.^21^ Considering the ED_50_ (64.7 mg) and t_1/2_ (3–7 hours) of caffeine, 92.17 mg of caffeine can occupy a substantial amount of A_2A_Rs, and it is possible that daily caffeine intake in the placebo group^21^ might already exert some symptomatic effects in PD. Therefore, further studies are required to investigate the symptomatic effects of caffeine in patients with PD who have no or less caffeine consumption. In addition, this study recommends measuring blood caffeine concentration or at least avoiding the consumption of caffeine‐containing products when investigating human A_2A_Rs.

In conclusion, this study shows that caffeine binds to striatal A_2A_Rs in a dose‐dependent manner. A sufficient A_2A_Rs occupancy can be obtained by drinking one cup of coffee, which is equivalent to approximately 100 mg of caffeine.

## Author Roles

K. Ishibashi, and K. Ishii designed the study. K. Ishibashi, Y.M., K.W., J.T., K. Ishiwata, and K. Ishii obtained the data. K. Ishibashi carried out the data processing. K. Ishibashi, and K. Ishii interpreted the data. K. Ishibashi drafted and revised the manuscript. All authors read and approved the final manuscript.

## Financial Disclosures of All Authors (for the Preceding 12 Months)

Kenji Ishibashi: Nothing to report.

Yoshiharu Miura: Nothing to report.

Kei Wagatsuma: Nothing to report.

Jun Toyohara: Nothing to report.

Kiichi Ishiwata: Nothing to report.

Kenji Ishii: Nothing to report.

## Data Availability

The data that support the findings of this study are available from the corresponding author upon reasonable request.
